# An international multi-stakeholder study on developing teacher capabilities and agency to implement and sustain physically active learning in schools

**DOI:** 10.3389/fspor.2026.1766380

**Published:** 2026-07-27

**Authors:** Andy Daly-Smith, Mathias Brekke Mandelid, Jade Morris, Emma Norris, Jouni Kallio, Hege Eikeland Tjomsland, Victoria Archbold, Tujia Tammelin, Amika Singh, Paula Silva, Jesper von Seelen, Caterina Pesce, Jo Salmon, John Bartholomew, Geir Kåre Resaland

**Affiliations:** 1 Research Institute for Health and Social Care, University of Bradford, Bradford, United Kingdom; 2 Centre for Physically Active Learning, Faculty of Education, Arts and Sports, Western Norway University of Applied Sciences, Sogndal, Norway; 3 Wolfson Centre for Applied Health Research, Bradford Royal Infirmary, Bradford, United Kingdom; 4 School of Sport, Leeds Beckett University, Leeds, United Kingdom; 5 Department of Pedagogy, Religion and Social Studies, Faculty of Education, Arts and Sports, Western Norway University of Applied Sciences, Bergen, Norway; 6 Health Behaviour Change Research Group, Brunel University of London, London, United Kingdom; 7 Likes, School of Health and Social Studies, JAMK University of Applied Sciences, Jyväskylä, Finland; 8 Mulier Institute, Utrecht, Netherlands; 9 Research Centre in Physical Activity, Health and Leisure, Faculty of Sport, University of Porto and Laboratory for Integrative and Translational Research in Population Health (ITR), Porto, Portugal; 10 Department for Research and Development, University College South Denmark, Haderslev, Denmark; 11 Department of Movement, Human and Health Sciences, University of Rome “Foro Italico”, Rome, Italy; 12 Institute for Physical Activity and Nutrition (IPAN), School of Exercise and Nutrition Sciences, Deakin University, Geelong, VIC, Australia; 13 Department of Kinesiology, Penn State University, University Park, PA, United States

**Keywords:** framework, movement-integration, multi-stakeholder, physically active learning, teacher training

## Abstract

**Background:**

Despite growing acceptance of integrating physically active learning (PAL) into schools, challenges remain regarding sustaining the results obtained in controlled experiments beyond the formal program. This study addresses this gap by exploring how in-service teacher training can be structured to support the sustained implementation of PAL.

**Methods:**

We adopted an international multi-stakeholder perspective and recruited teachers (*n* = 6), teacher educators (*n* = 6), and researchers (*n* = 6) to participate in online semi-structured interviews. All 18 stakeholders were recruited based on their expertise in PAL. Interviews were audio-recorded, transcribed, and analysed using reflexive thematic analysis.

**Results:**

We identified two overarching themes: (A) the process of gradual growth and (B) building a whole-school PAL culture. The first theme illustrates teachers’ gradual PAL growth through i) onboarding, ii) starting simple, and iii) building towards advanced practice. The second theme revolves around building a whole-school PAL culture and encompasses the process of sustaining PAL through (i) school and national policy and (ii) developing a PAL culture.

**Conclusions:**

Based on these themes, we developed a conceptual framework as a visual representation of the results structured around the phases of onboarding, starting simple, advanced practice, and building a whole-school PAL culture. The framework indicates that PAL teacher training is a comprehensive process that occurs in a synergistic relationship between teachers’ individual and collective agency to implement and sustain PAL in schools. This study and the proposed conceptual framework may help advance the implementation and sustainability of future PAL initiatives. However, the framework represents an interpretative synthesis of stakeholder perspectives and requires further empirical testing across educational contexts.

## Introduction

Physically Active Learning (PAL), defined as integrating movement within the delivery of academic content (1), has garnered global interest, as evidence suggests that physical activity can positively impact pupils’ academic performance (2, 3), cognitive processes (4, 5), and time on task (6). PAL has been positioned as a cost-effective approach to addressing core educational goals and increasing pupils’ physical activity levels during their most sedentary period of the day (7–9).

Despite growing interest, challenges remain in sustaining PAL beyond formal researcher-led programs (10–12). Existing programs are often predefined and lack flexibility for local adaptation, which can limit alignment with teachers’ professional practice and reduce long-term uptake (13, 14). Additionally, many programs are short-term, often consisting of brief training sessions, which may not sufficiently support sustained changes in teaching practice or help teachers overcome risk aversion (10, 15). As a result, PAL implementation remains confined to early adopters, limiting broader, scalable uptake across schools (8, 16–18).

In response, research has increasingly focused on identifying barriers and facilitators to PAL implementation (19, 20) and on developing more comprehensive approaches to teacher training in both pre-service and in-service contexts (1, 21–23). There has also been a growing emphasis on involving stakeholders in the design of such programs (8, 16). However, empirical knowledge on how to structure teacher training to support sustained PAL implementation remains limited (10, 24, 25).

To address this gap, the ACTivate project was established to improve PAL implementation and sustainment in schools. The project co-created a Europe-wide PAL teacher curriculum (22), an online training program, and a meta-synthesis of teachers’ perspectives on adapting and implementing PAL (1). In addition to revealing several themes on PAL, the synthesis also revealed that research on teacher training was scarce (1). Building on this foundation, this study aimed to explore how teachers, teacher educators, and researchers perceive the design of in-service training to support the implementation and sustainment of PAL in schools. Based on these perspectives, the study further develops a conceptual, heuristic framework that represents an interpretative synthesis of stakeholder experiences.

## Methodology

### Stakeholder identification and recruitment

To gain a global understanding of teacher training programs for implementing and sustaining PAL, we sought insights from three stakeholder groups: teachers, teacher educators, and researchers. Inclusion criteria comprised 1) in-service teachers in primary school with experience in PAL, 2) teacher educators who facilitated both pre-service and in-service teacher PAL training programs, and 3) researchers with a record of publishing PAL-related papers in peer-reviewed journals.

The recruitment process consisted of two phases. First, we purposively identified stakeholders affiliated with the ACTivate network who had contributed in a non-funded advisory capacity (26). These participants were familiar with the ACTivate project but were not directly involved. This initial phase resulted in the recruitment of four participants (1 teacher, 1 teacher educator, and 2 researchers). To broaden the range of perspectives beyond the ACTivate network, we subsequently employed a snowball sampling approach (27). Country leads within the ACTivate consortium were asked to identify relevant stakeholders across the three groups, and initial participants were invited to suggest additional stakeholders outside the consortium (27). While teachers and teacher educators were primarily identified through these referrals, researchers were primarily identified through their publication records. The process resulted in an additional 14 stakeholders.

Although participants varied in their background and experience, all were considered to have substantial experience with PAL (26). To enhance international diversity and avoid overrepresentation from any national context, we limited representation to approximately one stakeholder per group per country. In total, 18 participants were recruited, including six teachers, six teacher educators, and six researchers from eight countries (Table 1).

**Table 1:** Overview of stakeholders participating in the interviews.

Stakeholder group	Participants		Country represented	Interview duration	PAL years of experience
	Pseudonym	Gender			
Teachers	Harry^a^	Male	UK	53 minutes	>10 years
	Finn	Male	Norway	53 minutes	>10 years
	Isaak	Male	Finland	29 minutes	>10 years
	Sophie	Female	Netherlands	55 minutes	3 years
	Olivia	Female	USA	48 minutes	Not specified
	Emma	Female	Denmark	44 minutes	6 years
Teacher-educators	George^a^	Male	UK	51 minutes	8 years
	Alex	Male	Norway	74 minutes	>10 years
	Oscar	Male	Denmark	48 minutes	7 years
	James	Male	USA	43 minutes	>10 years
	William	Male	Netherlands	48 minutes	3 years
	Ava	Female	Finland	32 minutes	>10 years
Researchers	Leo	Male	UK	60 minutes	3 years
	Ella	Female	Finland	33 minutes	4 years
	Mason	Male	Australia	51 minutes	>10 years
	Lucas^a^	Male	USA	52 minutes	>10 years
	Amelia	Female	Ireland	38 minutes	7 years
	Rhys^a^	Male	Norway	42 minutes	6 years

Overview of included participants. All participants were assigned pseudonyms. Participants marked with ^a^ were purposively recruited through the ACTivate network, while the remaining participants were recruited through snowball sampling.

The sample size was considered sufficient to generate rich and diverse perspectives across stakeholder groups and national contexts, consistent with qualitative research principles that emphasise depth and contextual relevance over representativeness (26). Before data collection, all stakeholders were given information about the research purpose, the interview guide, and the requirements. Additionally, all stakeholders were asked to read and sign the consent form before participating. Ethical approval was granted by Leeds Beckett University (Ref 70882).

### Data collection

Due to the global recruitment, all interviews were conducted online via Zoom over a three-month period. The interview guide had a two-fold focus. The first part explored participants’ experiences, perceptions, and perspectives of PAL in practice. The second part was informed by the COM-B framework, which guided the formulation of questions related to participants’ perceptions of capability, opportunity, and motivation to implement and sustain PAL in school settings (28). Using COM-B as a sensitising framework for data collection, rather than for analysis, provided theoretical guidance while maintaining an inductive and interpretative analytical approach (26). In addition, the interview guide included questions on participants’ experiences with PAL, their involvement in training or implementation initiatives, and factors that may support the initial and long-term integration of PAL in schools. Particular attention was given to how PAL could be extended beyond early adopters and embedded within wider school practice. Follow-up questions were used to encourage all stakeholders to elaborate on examples, ideas, and concepts (26).

Interviews were conducted in English, Finnish, and Dutch. The Finnish interviews were conducted in Finnish and subsequently translated into English by a bilingual researcher (JK). The Dutch interviews were primarily conducted in English, with occasional translation support where needed. To support translation accuracy and preserve meaning, bilingual members of the research team (AS & JK) reviewed the translated transcripts. They engaged in collaborative discussions to ensure conceptual clarity and consistency across languages. Culturally specific terms and expressions related to PAL were carefully handled to maintain the integrity of participants’ perspectives. Following data collection, all participants were assigned pseudonyms, and identifying information, such as school names, was removed from the transcripts to ensure anonymity (26).

### Data analysis

We analysed the data using reflexive thematic analysis (RTA), as outlined by Braun and Clarke (29, 30). This approach was chosen for its flexibility in exploring stakeholders’ perspectives and generating interpretative insights aligned with the study aim. RTA was also chosen because it does not rely on reproducible coding procedures. Rather, it emphasises the researcher's active role in interpreting the data (29). The analysis followed the six phases: i) familiarisation with the data, ii) generating initial codes, iii) generating themes, iv) reviewing themes, v) defining and naming themes, and vi) writing the results.

First, transcripts were read repeatedly to develop familiarity with the dataset. During this phase, AD-S and JLM noted initial observations and interpretations. In the second phase, initial codes were generated inductively across the dataset. Coding was conducted collaboratively, with AD-S and JLM coding transcripts and discussing interpretations. Codes were treated as flexible and interpretative units, developed in relation to the study's aim. As such, codes were broad and captured a range of meanings, including concepts such as “local adaptation” and “PAL benefits”. A total of 62 codes were generated.

In the third and fourth phases, codes were refined and grouped into preliminary themes based on conceptual similarity and relevance to the study aim. Given our analytical focus on identifying shared patterns across the dataset, this process involved iterative movement between data, codes, and emerging themes. Overlapping and redundant codes were merged or removed, resulting in 46 refined codes. These were subsequently organised into six themes that broadly captured key aspects of the data: “PAL benefits”, “resources”, “delivery”, “capability”, “training”, and “building a whole school culture”.

In the fifth phase, the preliminary themes were further reviewed, refined, and synthesised. AD-S and MBM led this phase, working collaboratively to define and name themes that captured their underlying meaning while remaining grounded in the data (29, 30). Through this process, the initial themes were refined into sub-themes, and codes were condensed into broader, meaning-oriented categories. This phase also involved analysing broader patterns across the dataset, resulting in two overarching themes regarding the teacher training process (Table 2).

**Table 2:** Summary of the generated themes, sub-themes, and codes.

Theme	Sub-theme	Code
Process of gradual growth	Onboarding	Understanding teachers’ initial mindset Building teachers’ understanding of PAL through practice Helping teachers understand PAL benefits
	Starting Simple	Building teachers’ initial capability Building teachers’ initial agency
	Building towards advanced practice	Adopting a growth mindset Becoming a reflexive practitioner
Developing a whole-school culture	Policy advocacy	School and national policy
	Developing PAL culture	Building school leadership Peer support from colleagues Culture change

Throughout the analytic process, reflexivity was central. The research team comprised researchers with extensive experience in PAL and involvement with the ACTivate project, which informed our understanding of the field and may also have shaped our interpretations (29). To address this, the core team (AD-S, JLM, MBM, GKR) engaged in ongoing reflexive dialogue, critically examining assumptions and interpretations during the process. This collaborative effort allowed different perspectives within the research team to challenge and refine the final themes.

Finally, the sixth phase involved developing a coherent narrative linking the themes to the study's aim and the existing literature. MBM and AD-S drafted the initial version of the Results section, which was subsequently reviewed and refined by the wider authorship team (29).

## Results

Two overarching themes emerged from the analysis: (A) gradual growth and (B) building a whole-school PAL culture (Table 2). The first theme reflects teachers’ progression through three phases of PAL development: (i) onboarding, (ii) starting simple, and (iii) building towards advanced practice. The second theme captures the conditions for sustaining PAL through (i) alignment with school and national policy and (ii) the development of a whole-school PAL culture.

### Process of gradual growth

We developed the first theme to convey that gradual growth is an ongoing process across three phases: onboarding, starting simple, and progressing to become an advanced practitioner. This theme captures how stakeholders described the development of teachers’ capability and agency over time, including the adoption of a growth mindset and the development of reflexive practice.

#### Onboarding

The onboarding phase highlights the need to understand teachers’ initial mindset before aligning PAL with their educational values, beliefs and emotions. It was a process that extended beyond orienting them to the benefits of PAL. Instead, it was a process of guiding teachers to understand why and how PAL could be integrated into their practice.

##### Understanding teachers’ initial mindset

Stakeholders commonly described the importance of helping teachers relate PAL to their existing values by reflecting on its purpose and contribution. Supporting teachers in articulating their own understanding of PAL was seen as a key initial step in the onboarding process. This was reflected in the view that “*It needs to be meaningful regarding their pedagogical approach*” (Oscar, teacher educator). Oscar alluded to teachers’ professionalism and the idea that supporting them through pedagogical reasoning could help establish an initial PAL mindset.

Similarly, other participants emphasised the importance of connecting PAL to teachers’ beliefs and underlying assumptions of education: “*asking the right questions can help them connect with their inner beliefs”* (Rhys, researcher). Several stakeholders suggested that discussing teachers’ fundamental perspectives on education and children could improve their understanding of PAL's potential. One teacher educator noted that “*PAL becomes engaging when it becomes emotional*” (James), indicating that affective dimensions were also considered important in shaping initial engagement. Taken together, these accounts suggested that onboarding involves supporting teachers in developing a meaningful understanding of PAL that aligns with their pedagogical values, beliefs, and emotions.

##### Building teachers’ understanding of PAL through practice

Stakeholders also described the importance of supporting teachers’ understanding of PAL through practical experience. On this point, stakeholders suggested that for teachers to recognise the value of PAL, they need to see how it can be integrated in everyday teaching contexts:

“I often think about one situation where a teacher said: ‘What is the point of PAL? Later, when she came to watch the session, she turned to me and said, ‘I get it. The children are having fun and are engaged, and it seems like the learning is sticking” (George, teacher educator).

In line with this account, several participants described teachers as primarily practical and experiential learners, highlighting the value of observing and trying PAL in practice. Stakeholders suggested that experiencing PAL firsthand could help teachers recognise its relevance and reflect on potential barriers and opportunities. One teacher articulated this view: “*Teachers must try it out for themselves. It helps them embody the essence of PAL and realise that they might already be doing something similar”* (Finn, teacher). Across accounts, participants highlighted that aligning PAL with existing teaching practices could help teachers recognise its opportunities and relevance in their own contexts.

##### Helping teachers understand PAL benefits

The third and final aspect of the onboarding phase involved developing an understanding of the value of PAL. Stakeholders described this as an important factor in shaping teachers’ motivation and engagement. As one researcher noted: “*Trying to change teachers’ perceptions by getting them to believe its value (…) is half of the battle”* (Leo). Stakeholders commonly related these benefits to pupils’ engagement and enjoyment, health outcomes, and classroom behaviour. However, more pressing was ensuring teachers felt that PAL contributed to pupils’ learning and broader educational goals. Getting acquainted with the benefits of PAL served a dual role: both illustrating its effectiveness and giving teachers a clear idea of what they could achieve: “*I think that teachers need practical perspectives, as well as the research and science behind it”* (Harry, teacher). Together, these accounts suggest that onboarding involves supporting teachers in recognising both practical and theoretical dimensions of PAL, so that they can understand the scope of its application and the potential opportunities it offers.

#### Starting simple

The second phase in the process of teachers’ gradual growth was described as starting simple. This phase reflects how stakeholders emphasised the importance of developing teachers’ initial capability and agency, enabling them to independently continue experimenting with PAL in their practice.

##### Building teachers’ initial capability

Stakeholders commonly described teachers’ capability as central to implementing and sustaining PAL, referring to their ability to enact PAL within their own teaching practice. To support this, stakeholders emphasised the importance of starting with simple, accessible approaches, such as starter packs, resources, materials, or examples from other teachers. Moreover, this could also include concrete examples of the requirements for integrating PAL into their teaching. These were described as entry points that could support teachers in understanding how PAL could be integrated into their teaching. One stakeholder explained: “*Try and explain what PAL is, and usually start quite early with something practical so they can see what we mean by it*” (Amelia, researcher). Many stakeholders also highlighted that starting with practical examples could help teachers recognise connections between PAL and their existing pedagogical approaches. This was further illustrated by one teacher educator who exemplified that:

“All teachers have some competence they can draw on. Suppose it is not high-intensity activities but drama, games, or outdoor activities. They often realise they have relevant experience and then go, ‘Oh, maybe I do know PAL after all’” (Oscar).

These accounts suggest that building initial capability involves supporting teachers in recognising and drawing on their existing skills, knowledge and experiences. Stakeholders further described how this process also involves adapting and refining familiar activities to align with PAL, thereby allowing teachers to integrate it into their current practice and to suit their particular needs. In this way, stakeholders notably argued that PAL could initially be experienced as an add-on to traditional teaching. However, becoming aware of initial capability could increase teachers’ confidence in using PAL and support the perception that PAL could become a seamless part of their teaching practice.

##### Building teachers’ initial agency

Building teachers’ agency was another key aspect of the theme ’starting simple’. Stakeholders commonly associated agency with teachers’ sense of freedom and responsibility for planning and organising good-quality teaching. While closely related to capability. Agency was described as the ability to use these capabilities confidently and flexibly in practice. Stakeholders highlighted that this could be challenging, as they perceived that some teachers are hesitant to take risks in contexts because of how they are evaluated based on pupil outcomes. One researcher noted that: “(…) *teachers do not necessarily want to take risks in the subject where they are judged on pupils’ performance (…) by their principal, national metrics and so on”* (Rhys). In response, stakeholders described the importance of supporting teachers so they feel confident and safe to experiment with PAL, even when outcomes are uncertain. Building an initial agency was thus characterised as enabling teachers to apply their capabilities while developing confidence in their own professional judgement.

Capability was essential to building initial agency, as stakeholders described that it could provide teachers with the core skills and confidence to use PAL and integrate it with conscious reflection on its purpose. Some stakeholders also suggested that this process involved becoming “*able to adapt and become more autonomous continuously” (*Amelia, researcher), thereby reflecting a gradual shift towards more independent and reflective practice. This process was further illustrated by one teacher's account of their own experience: “*So I started discussing with the principal. I asked him to change the timetable because I do not want PAL to be fixed and rigid.* (…) *I want the freedom to integrate PAL when it is natural.”* (Finn). This example illustrates how, over time, teachers may develop their own perspectives on integrating and sustaining PAL in their contexts. Although Finn felt it was easier to start in a rigid way by having a school leader put PAL on the schedule, his initial capability and agency helped him reflect on when and where it was natural to integrate PAL. Taken together, these accounts suggest that developing initial agency involved supporting teachers in recognising their capacity to act and gradually building their confidence in using their professional judgement.

#### Building towards advanced practice

The third phase of gradual growth reflects stakeholders’ descriptions of a progression towards more advanced and integrated use of PAL. This phase was characterised by the development of a growth mindset, in which teachers are increasingly engaged in ongoing cycles of planning, experimenting, and reflecting on their use of PAL.

##### Adopting a growth mindset

Stakeholders associated the development of a growth mindset with teachers’ beliefs in their ability to continuously develop their capability and agency in relation to PAL. This perspective was linked to the view that there are no clearly demarcated levels of PAL competence and that development is an ongoing process throughout teachers’ professional lives. As a teacher educator noted: “*Teachers need to accept the fact that there will be hiccups, bumps and scrapes along the way*” (George). Similarly, stakeholders also described how engaging with PAL involves a willingness to experiment and learning from a continuous process of trying and failing: “*You need to be open-minded and continue trying different approaches, new stuff, new equipment and maybe new alternatives to teaching without knowing if they will work”* (Finn, teacher). These accounts suggest that a growth mindset involves openness to trying and refining different approaches, and recognising that challenges and setbacks are part of the process. Some stakeholders also described how this process could support teachers in developing confidence and capability in integrating PAL into lessons. In addition, stakeholders associated a growth mindset with engaging in ongoing cycles of planning, acting, and reflecting, even when initial attempts were not successful.

##### Becoming a reflexive practitioner

Stakeholders further described that progressing towards advanced practice involved becoming increasingly reflexive in integrating PAL into teaching. This included moving beyond viewing PAL as an add-on to fully integrating it across all subjects and learning activities. For these reasons, many stakeholders associated teachers’ reflexivity with their ability to move away from their lesson plan and support new and unexpected situations:

“One aim is learning, for instance, language. The other aim is physical activity. So, throw a ball. The art of PAL is combining the two meaningfully. However, that is not for beginners, as it requires active reflection on your actions” (Sophie, teacher).

Across stakeholders, a common perspective was the importance of continuously reviewing the integration of PAL, recognising that approaches may not function in the same way across contexts or over time. For some, this ability meant reviewing the activity afterwards, reflecting on its purpose and why it did not work. For others, it meant continuously reviewing during: *“Another thing I found helpful was becoming a facilitator. Sometimes, I know what the rules are supposed to be, but then the kids take it in a different direction. So, I let them go with it”* (Olivia, teacher). In this quotation, Olivia illustrates the core of becoming a reflexive practitioner. That is, teachers’ ability to review and adjust the activity before and during teaching. These examples illustrate how reflexivity was associated with teachers’ ability to review, adapt, and respond to different classroom situations. Stakeholders also linked this to teachers’ attentiveness towards the pupils’ needs and prerequisites. Linking to this was also a perception that reflexive teachers are better at evaluating existing and creating new activities:

“*I look at my colleagues, and I see ideas flowing. They are not always new ideas, but alternatives. They seem to develop a single basic activity and refine it with variations. It may sound like a new idea, but they apply it in new classes with different levels to benefit more pupils*” (Sophie, teacher). Taken together, these accounts suggest that becoming a reflexive practitioner involves developing the ability to integrate PAL flexibly and in context-responsive ways, supported by ongoing reflection and adaptation.

### Developing a whole-school PAL culture

The second overarching theme reflects stakeholders’ perceptions that sustaining PAL extends beyond individual teacher development and is closely linked to broader organisational and policy-related conditions. Participants described how individual growth may be more sustainable when aligned with a collective whole-school PAL culture. Within this theme, two sub-themes were identified: (i) policy advocacy and (ii) PAL culture.

#### Policy advocacy

The sub-theme of policy advocacy captures how stakeholders described policy's role as a structural influence on the integration and sustainment of PAL in schools. Stakeholders commonly referred to both national and school-level policies as shaping the extent to which PAL is prioritised and supported within educational settings.

##### School and national policy

Stakeholders described school policies as influencing teachers’ opportunities to implement PAL. As a researcher noted: “*I do not know if policy is the right word, but it is something about what gets adopted in schools.* (…) *Teachers do what they think the school perceives as important”* (Leo). The quotation reflects how some stakeholders perceived schools’ priorities as an important factor in shaping teachers’ practices and the long-term sustainment of PAL. Several stakeholders additionally highlighted the role of school leaders in interpreting and embedding broader educational priorities into local policies and practices. School policy was thus perceived as closely intertwined with national policy, as it enabled broader approaches to teaching and learning.

While school policies were often described as enabling, stakeholders raised concerns about national-level policies. Across national contexts, stakeholders highlighted that the curriculum was perceived as demanding, with limited time and space for experimenting with approaches such as PAL. As one researcher noted: “*Based on my experience, there are many teachers who want to do PAL (…), but the system is hampering their implementation”* (Rhys). The system in this case was the curriculum demands, international test regimes, and learning expectations. Although stakeholders argued that educational policies could be agnostic about teachers’ teaching styles, they were particularly concerned that accountability systems demanding clear evidence of progression could be at odds with teachers’ agency in sustaining PAL. As such, some stakeholders argued that national policies comprised a significant reason why some teachers viewed PAL as a risk. Others, however, argued that schools had to be conscious of how to navigate the policies to help teachers sustain PAL as part of their school culture.

In addition to educational policies, stakeholders also noted that national health policies influenced how teachers interact with PAL. Some stakeholders described these policies as promoting physical activity in ways that were not always aligned with the school context: “(…) *School strategies to make that 60 minutes are difficult, they [government] went, ‘Right, we will raise the bar even more’, and schools went, ‘We cannot do this’*” (Mason, researcher). In line with this account, stakeholders suggested that while they often found such policies well-intentioned, they were sometimes experienced as top-down and lacking contextual sensitivity to what the school could grasp. Additionally, some stakeholders described how educational priorities were often perceived as taking precedence over health-related objectives. One stakeholder noted that: “*From my experience, teachers want to do PAL, but they are always evaluated on learning objectives, and they cannot be bothered with health. The system is hampering their integration of PAL”* (Rhys, researcher).

Taken together, these accounts highlight how stakeholders perceived policy environments as both enabling and constraining factors in sustaining PAL, shaping how it is prioritised and enacted within schools. For these reasons, stakeholders argued that it was important for schools to understand education and health policies to navigate priorities and incorporate them into local school policies.

#### Developing a PAL culture

The final sub-theme reflects how stakeholders described the transition from individual efforts to a more collective, whole-school approach to sustaining PAL. Stakeholders emphasised that involving the wider school community was important in supporting long-term integration. This sub-theme comprised three interrelated aspects: school leadership, peer support, and broader culture change.

##### Building school leadership

Stakeholders commonly emphasised that school leadership was crucial to establishing and shaping a PAL culture. School leaders were described as influential in setting priorities, supporting initiatives, and shaping the conditions under which PAL could be implemented. A teacher educator even said that: “*where the principal comes along in their leggings and their trainers for the CPD session, the odds are better with those schools”* (George). In line with George's quotation, the general perspective was that when school leaders were actively involved in PAL-related activities, it was easier to establish a shared commitment within the school. Stakeholders also described how leadership engagement could support teachers by legitimising PAL practice and embedding them within everyday school routines.

These descriptions further highlighted connections between school leadership and policy-related structures, where school leaders were seen as influencing scheduling, resource allocation, and the organisation of professional learning environments. Together, these accounts suggest that school leadership was perceived as an important factor in fostering a whole-school PAL culture.

##### Peer support from colleagues

Stakeholders also emphasised the role of peer support as essential to increasing the likelihood of successfully sustaining PAL in schools, as it could help establish a whole-school culture and engage mid- and late adopters. Indeed, many stakeholders related peer support to the professional learning environment and the school's collective ability to evolve and sustain PAL. Peer support was described as occurring through both informal interactions and more structured professional collaboration. While informal exchanges were valued, some stakeholders highlighted the potential of more structured formats for reflection and feedback: “*We want them to reflect upon practice (…) We want to structure teachers’ feedback so they can be critical, constructive”* (Alex, teacher educator). By providing critical feedback, stakeholders argued that these forms of interactions could enable teachers to share experiences, discuss challenges, and collectively develop solutions: “*If teachers talk about barriers with other teachers, they are often able to solve the problem”* (Amelia, researcher). In line with this quotation, some stakeholders further emphasised that peer interactions could support the development of shared practices over time: “*We should not undermine that teachers are resourceful (…) if we expose enough teachers to these teaching methods [PAL], it will become the norm”* (Lucas, researcher). Notably, the stakeholder outlined that discussing the problems was an essential first step, but also emphasised that discussions could be accompanied by guiding questions to help teachers elevate their reflections and perspectives beyond their own experiences.

Taken together, these accounts suggest that peer support was perceived as contributing to the development of collective agency, where teachers collaboratively shape and sustain PAL practices. Stakeholders viewed peer support as essential to building a culture where teachers’ collective agency could sustain PAL.

##### Culture change

Stakeholders described the development of a whole-school PAL culture as involving broader changes beyond individual or small-group initiatives. For instance, some stakeholders noted that focusing on a small group of teachers could limit broader PAL integration. One stakeholder explained that: “*By only focusing on the 3rd, 4th, and 5th grades, I am basically saying that this is unimportant because I am not asking the entire school to do PAL”* (Lucas, researcher). Across accounts, stakeholders described whole-school engagement as supporting a more coherent and sustained approach to PAL. This was linked to collaboration, shared professional development, and alignment across different levels of the school. One particular teacher educator reflected on this broader perspective to a whole-school engagement: “*Then it is only 30-40% about PAL. The rest is about learning to work as an individual, with a team, with the entire school system”* (Alex). This quotation alludes to using PAL to challenge established teaching methods and change the culture, thereby supporting teachers to develop a learning environment.

In further accordance with the above-mentioned quotation, stakeholders also described how developing a PAL culture involves negotiating the relationship between individual freedom and collective goals: “*We should not overlook that teaching is a highly autonomous job where they get to decide what they do (…). So principals must not undermine their [teachers] competence”* (Lucas, researcher), thereby highlighting the importance of recognising teachers’ professional judgement. Similarly, another stakeholder described how leadership and teacher voices could interact in shaping sustainment: “*successful whole-school implementation is where they have a combined approach where you have the school leadership guiding the way, putting PAL on the schedule while allowing teachers to raise their voice about how to sustain it”* (Rhys, researcher). Taken together, these accounts suggest that developing a whole-school PAL culture involves ongoing interactions between leadership, collaboration, and teacher autonomy, shaping how PAL is discussed, adapted, and sustained within schools.

## Discussion

**Figure 1:**
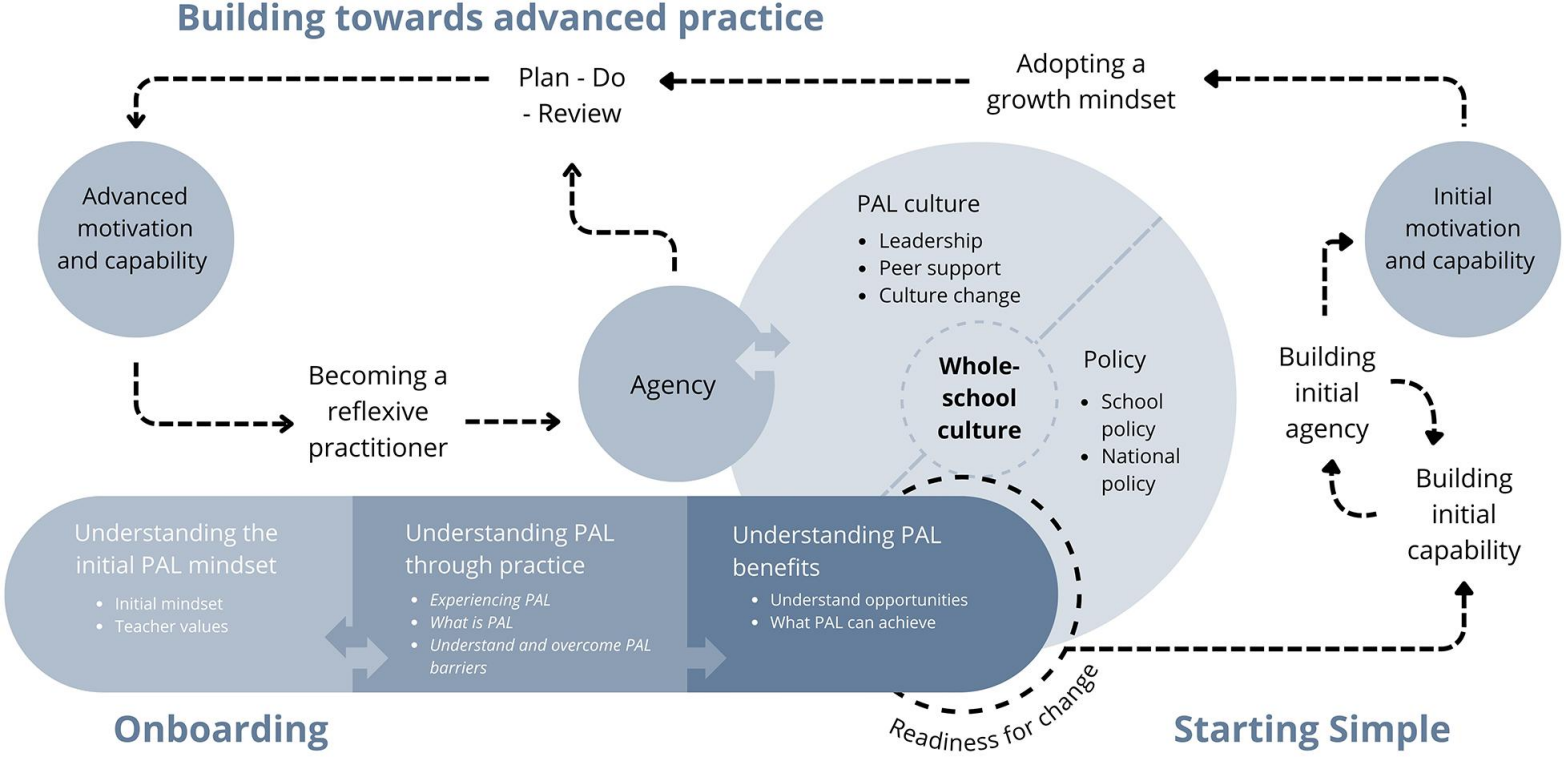
The PAL teacher training framework.

The international multi-stakeholder perspective adopted in this study provides novel insights into how PAL capability and agency can be developed across educational contexts, advancing beyond single-stakeholder perspectives and individual theoretical approaches (14, 16, 23, 25). In this section, we synthesise the results by presenting a conceptual, heuristic framework for PAL teacher training (Figure 1). The framework is structured around the three stages of gradual growth: i) onboarding, ii) starting simple, iii) advanced practice, alongside a fourth phase related to developing a whole-school PAL culture. Together, the results suggest that PAL teacher training could benefit from moving beyond short-term approaches, such as one-day training programs (10, 24), towards more sustained and contextually responsive models. When considered alongside existing research on teacher motivation (17, 18, 31), capability (32–34), and implementation challenges (14, 23, 25), the framework provides a novel contribution that can inform future PAL teacher training initiatives.

First, the framework begins with an often underexplored phase, ‘onboarding’, in which PAL is aligned with teachers’ educational values, beliefs, and existing practices. Rather than focusing on predefined strategies or generalised implementation models (9, 10), the onboarding phase emphasises sensitivity to teachers’ contexts and needs. This aligns with previous research indicating the importance of school ethos and local contexts in shaping the implementation process (15, 16, 21). Within the framework, sensitivity refers to recognising the diversity of school environments and drawing on the perspectives of school leaders, teachers, and pupils (8, 16, 35). In this regard, implementation may benefit from engaging with teachers’ daily practice and the practical challenges they encounter, thereby supporting their readiness for change and willingness to experiment with PAL (15, 24). Although this study primarily relates context sensitivity to the first phase, previous research suggests that such considerations are relevant across all stages of implementation (35).

The second phase builds teachers’ initial confidence to enact PAL. As illustrated in Figure 1, the phase of starting simple reflects the early development of teachers’ capabilities and agency. In this study, building capability refers to teachers’ skills and knowledge for enacting PAL. Consistent with previous research, this involves strengthening teachers’ ability to make pedagogical choices, adapt activities, and further develop PAL in their own practice (32, 33).

The study further suggests a close relationship between capability and agency. Drawing on stakeholders’ accounts, teacher agency is understood as teachers’ capacity to act purposefully and make professional judgements within the structural and contextual conditions of their work. This aligns with previous research, which conceptualises agency as a relational and context-dependent capacity that develops over time (36, 37). Although often associated with autonomy or motivation, agency is conceptually distinct in that it emphasises teachers’ ability to interpret, negotiate, and respond to their professional context (36). Supporting the development of agency may be important as previous research has identified a lack of confidence and inclusion as barriers to sustaining PAL (19, 20, 31).

As such, the study suggests that capability supports the emergence of agency. As teachers gain initial skills, they are perhaps more willing to act, experiment with PAL, and adapt their practices to local conditions. While this aligns with previous research (13, 18, 38), the framework developed in this study highlights how this process may extend over time towards more reflexive and contextually responsive forms of practice, including negotiating constraints, taking pedagogical risks, and contributing to collective efforts within the school. This progression suggests that sustaining PAL depends not only on building skills but also on supporting teachers’ capacity to act within their professional context.

As further illustrated in the proposed framework (Figure 1), the third phase of becoming an advanced and reflexive practitioner involves continuously planning, trialing, and reviewing the use of PAL across different contexts. Consistent with previous research, this study indicates that it is a complex, time-consuming process involving trial and error, in which teachers persist despite initial challenges (1, 32). The third phase adds to existing literature by moving beyond the notion of clearly demarcated levels of PAL capability (33). Instead, it highlights that sustaining PAL likely involves developing reflexivity and a growth mindset as part of an ongoing professional process. These qualities appear closely connected to agency, as they enable teachers to make informed and contextually responsive pedagogical decisions (15, 34, 37).

The fourth phase focuses on developing a whole-school PAL culture. In line with the broader literature, whole-school culture can be understood as the development of shared norms, values, and practices (39). In this study, a whole-school culture was reflected in shared understandings of PAL's role. The proposed framework underscores that policy and organisational contexts may influence the implementation of PAL, as curriculum demands, accountability structures, and competing priorities shape local conditions. These factors have been widely identified as barriers in the PAL literature (19, 20). However, rather than treating these factors solely as constraints, the fourth phase of the framework emphasises how schools can actively interpret and translate national and international policy in ways that align with their local ethos. Aligned with previous research, school leaders play a key role in fostering a whole-school PAL culture through interactions with colleagues and by supporting alignment between individual practices and collective priorities across the school (40).

These processes may be understood as interactions between individual and collective forms of agency (15, 37). As teachers develop more advanced practices, these could help them articulate and shape shared approaches to sustaining PAL within their school context. Consistent with previous research, these results indicate that while school leadership plays an important role in prioritising PAL, implementation and sustainment appear to be collaborative efforts to translate individual experience into shared school knowledge (8, 39, 41). As such, the development of a whole-school PAL culture likely supports the school's collective capacity to sustain PAL by moving beyond reliance on individual teachers’ engagement (14, 15).

While the framework is conceptual, it could be used to prompt consideration of practical implementation aspects. For example, it may guide teachers to iteratively trial and adapt PAL in their everyday practice to navigate challenges and refine their approaches to integrating movement into teaching. Moreover, the framework highlights how leadership practices can support the integration of PAL into school policy and everyday routines. In this regard, school leaders could play a complementary role by interpreting and aligning national policy with local school priorities, thereby contributing to long-term sustainability (42). This would be particularly important as previous research has identified awareness of national policies as central to the adaptation of PAL into local school contexts (8, 43).

In addition, the framework draws attention to how teacher training programs can be structured in practice. Rather than providing a prescriptive model, it highlights that implementing and sustaining PAL is likely to require time and ongoing engagement. Although aspects such as program duration, delivery modes (e.g., workshops, school-based professional learning) and support mechanisms were recognised as important, they were not examined in depth in this study. As these factors will likely shape how the framework is operationalised across different contexts, they warrant attention in future research. The proposed framework may therefore offer a useful basis for teacher educators and program designers seeking to support both individual and collective processes of PAL implementation.

## Strengths and limitations

This study offers an international, multi-stakeholder perspective on the development of PAL teacher training, contributing a conceptual framework that outlines key phases of the implementation and sustainment. At the same time, several limitations need to be acknowledged. One consideration relates to sample size and data saturation. Within RTA, the aim is not to achieve saturation in a positivist sense, but rather to generate rich, meaningful, and contextually grounded insights (29). The sample size of 18 international stakeholders was considered sufficient to capture a diverse range of stakeholder perspectives across contexts (26, 29). However, the use of purposive and snowball sampling, including recruitment through the ACTivate network, could have influenced the perspectives represented. Stakeholders were highly engaged and experienced with PAL, which may mean that the results reflect the views of early adopters and do not fully capture the perspectives of less-experienced teachers or schools. This could affect the transferability of the results, as including different or less engaged stakeholders may have led to alternative interpretations.

A further limitation relates to researcher positionality. Several members of the research team have prior experience in PAL and involvement in the ACTivate project. This may have influenced both data collection and interpretation. To minimise potential influence and bias, interviews were conducted using open-ended questions and encouraged stakeholders to share both supportive and critical feedback. In addition, the research team engaged in reflexive dialogues throughout data collection and analysis to examine assumptions and interpretations. The multilingual nature of the data also represents a potential limitation. As some interviews were conducted and translated across languages, nuances in meaning could have been influenced despite efforts to ensure conceptual accuracy through bilingual review and collaborative verification.

Together, the results can be understood as an interpretative synthesis of stakeholders’ perspectives rather than an exhaustive account. Accordingly, the proposed framework is heuristic and represents a conceptual and visual synthesis of the study results (44). Consequently, our results might not transfer directly to all contexts. Instead, they offer guiding principles that likely require local adaptation. It is also important to recognise that comprehensive frameworks, such as the one presented in this study, can present practical challenges (19, 20). For example, extensively designed training initiatives risk being perceived as time-consuming or demanding for teachers (21, 25). While the results in this study suggest moving beyond short-term training formats, such as one-day training programs, this study does not provide specific guidance on program duration or intensity.

As such, the framework is an initial, conceptual model that has not yet been empirically tested or evaluated. It represents a heuristic synthesis of stakeholders’ perspectives and not a validated or prescriptive approach to implementation and sustainment. Although the framework is organised into four phases, these are overlapping and iterative. Further empirical research is therefore needed to examine the framework's applicability and effectiveness across contexts. For example, future studies should test the framework by trialing different phases within teacher training programs and evaluating their impact on teachers’ capability and agency, as well as the sustained use of PAL over time. Longitudinal and mixed-methods studies would be valuable in these settings as they can support the exploration of how the framework operates in practice, including its feasibility, duration, and adaptability across different schools and national contexts. In addition, it is important for future research to explore how schools can be supported in evaluating and sustaining change over time, including the development of tools for reflection and self-evaluation. Finally, consistent with previous research, the implementation and sustainment of PAL is likely to benefit from collaboration among multiple stakeholders, including teachers, school leaders, and external facilitators (15).

## Conclusion

This study offers new insights into how PAL teacher training could be evolved to support the implementation and sustainment of PAL in schools. The proposed framework provides a conceptual and heuristic synthesis of stakeholders’ perspectives, contributing to ongoing efforts to better understand the development of teachers’ capability and agency. Taken together, the framework's three phases, which focus on teacher growth, recognise that change is a complex and time-consuming process that benefits from sensitivity and responsiveness to the needs of various staff and schools. The fourth phase of building a whole-school PAL culture reinforces the school's importance in establishing more sustainable practices that move PAL implementation beyond isolated classrooms or lone teachers. As such, the framework provides a useful basis for informing future PAL initiatives. Researchers, teacher educators, teachers, and school leaders could consider exploring the framework's applicability across national and local contexts. Further empirical work is needed to examine how the framework can support the implementation and sustainment of PAL over time.

## Data Availability

The raw data supporting the conclusions of this article will be made available by the authors, without undue reservation.

## References

[1] Daly-Smith A Morris JL Norris E Williams TL Archbold V Kallio J. Behaviours that prompt primary school teachers to adopt and implement physically active learning: a meta synthesis of qualitative evidence. Int J Behav Nutr Phys Act. (2021) 18(1):1–20. 10.1186/s12966-021-01221-9 34801039 PMC8605507

[2] Barbosa A Whiting S Simmonds P Scotini Moreno R Mendes R Breda J. Physical activity and academic achievement: an umbrella review. Int J Environ Res Public Health. (2020) 17(16):5972. 10.3390/ijerph17165972 32824593 PMC7460146

[3] Singh A Saliasi E van den Berg V Uijtdewilligen L Groot R Jolles J. Effects of physical activity interventions on cognitive and academic performance in children and adolescents: a novel combination of a systematic review and recommendations from an expert panel. Br J Sports Med. (2018) 53:640. 10.1136/bjsports-2017-098136 30061304

[4] Hillman CH Erickson KI Kramer AF. Be smart, exercise your heart: exercise effects on brain and cognition. Nat Rev Neurosci. (2008) 9(1):58. 10.1038/nrn2298 18094706

[5] Lubans D Richards J Hillman C Faulkner G Beauchamp M Nilsson M. Physical activity for cognitive and mental health in youth: a systematic review of mechanisms. Pediatrics. (2016) 138(3):3. 10.1542/peds.2016-1642 27542849

[6] Norris E Steen T Direito A Stamatakis E. Physically active lessons in schools: a systematic review and meta-analysis of effects on physical activity, educational, health and cognition outcomes. Br J Sports Med. (2019) 54:826. 10.1136/bjsports-2018-100502 31619381

[7] Bartholomew JB Jowers EM. Physically active academic lessons in elementary children. Prev Med. (2011) 52:S51–4. 10.1016/j.ypmed.2011.01.017 21281672 PMC3116963

[8] Daly-Smith A Quarmby T Archbold VSJ Routen AC Morris JL Gammon C. Implementing physically active learning: future directions for research, policy, and practice. J Sport Health Sci. (2020) 9(1):41–9. 10.1016/j.jshs.2019.05.007 31921479 PMC6943765

[9] Webster CA Beets M Weaver RG Vazou S Russ L. Rethinking recommendations for implementing comprehensive school physical activity programs: a partnership model. Quest. (2015) 67(2):185–202. 10.1080/00336297.2015.1017588

[10] Vazou S Webster CA Stewart G Candal P Egan CA Pennell A. A systematic review and qualitative synthesis resulting in a typology of elementary classroom movement integration interventions. Sports Med Open. (2020) 6(1):1. 10.1186/s40798-019-0218-8 31907711 PMC6944721

[11] Lander N Mazzoli E Cassar S Symington N Salmon J Lander N. Embedding active pedagogies within pre-service teacher education: implementation considerations and recommendations. Children. (2020) 7(11):207. 10.3390/children7110207 33147706 PMC7692750

[12] Mandelid MB. Approaching physically active learning as a multi, inter, and transdisciplinary research field. Front Sports Act Living. (2023) 5:1–8. 10.3389/fspor.2023.1228340 PMC10416638 37576609

[13] Lander N Mazzoli E Essiet IA Telford A Ridley K Symington N. Equipping future teachers with innovative strategies that increase physical activity in the classroom: a hybrid implementation trial across three Australian Universities. Front Educ. (2023) 8:1093234. 10.3389/feduc.2023.1093234

[14] Donnelly JE Hillman CH Castelli D Etnier JL Lee S Tomporowski P. Physical activity, fitness, cognitive function, and academic achievement in children. Med Sci Sports Exerc. (2016) 48(6):1197–222. 10.1249/mss.0000000000000901 27182986 PMC4874515

[15] Hofmann R. The four paradoxes that stop practitioners from using research to change professional practice and how to overcome them. Educ Sci. (2024) 14(9):996. 10.3390/educsci14090996

[16] Routen A Johnston J Glazebrook C Sherar L. Teacher perceptions on the delivery and implementation of movement integration strategies: the CLASS PAL (physically active learning) programme. Int J Educ Res. (2018) 88:48. 10.1016/j.ijer.2018.01.003

[17] Lerum Ø Eikeland Tjomsland H Leirhaug PE McKenna J Quaramby T Bartholomew J. The conforming, the innovating and the connecting teacher: a qualitative study of why teachers in lower secondary school adopt physically active learning. Teach Teach Educ. (2021) 105:103434. 10.1016/j.tate.2021.103434

[18] Mwaanga O Dorling H Prince S Fleet M. Understanding the management challenges associated with the implementation of the physically active teaching and learning (PATL) pedagogy: a case study of three isle of Wight primary schools. Managing Sport and Leisure. (2018) 23(4–6):408–21. 10.1080/23750472.2019.1568906

[19] Nathan N Elton B Babic M McCarthy N Sutherland R Presseau J. Barriers and facilitators to the implementation of physical activity policies in schools: a systematic review. Prev Med. (2018) 107:45–53. 10.1016/j.ypmed.2017.11.012 29155228

[20] Riley N Mavilidi M Kennedy S Morgan P Lubans D. Dissemination of thinking while moving in maths: implementation barriers and facilitators. Translational Journal of the ACSM. (2021) 6:e000148. 10.1249/TJX.0000000000000148

[21] Mandelid MB Dyngeland ES Teslo S Lerum Ø Tjomsland HE Jenssen ES. Workplace-based continuing professional development program for physically active learning: designing a framework and prospective directions. Front Educ. (2024) 9:1407542. 10.3389/feduc.2024.1407542

[22] Daly-Smith A Ottesen CL Mandelid MB Von Seelen J Trautner N Resaland GK. ACTivate European Physically Active Learning Teacher Training Curriculum. Norway: Western Norway University of Applied Sciences (2021). p. 1–31. 10.13140/RG.2.2.34546.99529

[23] Lander N Patterson K Lai SK Martin-Alcaide N Orr J Weatherell N. From theory to practice: examining the influence of physically active learning on curriculum design and pedagogical planning in initial teacher education. Front Sports Act Living. (2025) 7:1719341. 10.3389/fspor.2025.1719341 41384143 PMC12689541

[24] Teslo S Jenssen ES Thurston M Mandelid MB Resaland GK Chalkley A. It’s the journey, not the arrival that matters – teachers’ perceptions of their practice after participating in a continuing professional development program in physically active learning. Teach Teach Educ. (2023) 136:104377. 10.1016/j.tate.2023.104377

[25] Naylor P Nettlefold L Race D Hoy C Ashe M Higgins J. Implementation of school based physical activity interventions: a systematic review. Prev Med. (2015) 72:95. 10.1016/j.ypmed.2014.12.034 25575800

[26] Creswell JW. Research Design: Qualitative, Quantitative & Mixed Methods Approaches. 5th edn Los Angeles, California: SAGE Publications, Inc. (2018) (Qualitative, quantitative and mixed methods approaches).

[27] Parker C Scott S Geddes A. “Snowball sampling”. In: Atkinson P Delamont S Cernat A Sakshaug J Williams R, editors. Research Design for Qualitative Research. London: SAGE Publications Ltd (2019). 10.4135/9781526421036831710

[28] Michie S van Stralen MM West R. The behaviour change wheel: a new method for characterising and designing behaviour change interventions. Implement Sci. (2011) 6(1):1. 10.1186/1748-5908-6-42 21513547 PMC3096582

[29] Braun V Clarke V. Reflecting on reflexive thematic analysis. Qual Res Sport Exerc Health. (2019) 11(4):589. 10.1080/2159676x.2019.1628806

[30] Braun V Clarke V. Thematic Analysis: A Practical Guide. Los Angeles: SAGE (2022).

[31] Knudsen LS Skovgaard T Bredahl TVG. ‘I like it’: exploring teachers’ motivation for using classroom-based physical activity in Danish public schools from a self-determination perspective. Results from a mixed methods study. Teach Teach Educ. (2021) 106:103439. 10.1016/j.tate.2021.103439

[32] Mandelid M Johansen JM Resaland GK Bratland-Sanda S Daly-Smith A Tjomsland HE. Beyond borders: developing the core aspects of physically active learning enactment (CAPAbLE) model in the third space. Scand J Educ Res. (2026) 70(1):130–46. 10.1080/00313831.2025.2459905

[33] Moon J Webster C. MI (my) wheelhouse: a movement integration progression framework for elementary classroom teachers. J Phys Educ Recreat Dance. (2019) 90:38–45. 10.1080/07303084.2019.1644258

[34] Madsen K. Pedagogy of movement integration – a threefold pedagogy grown through action research. Pedagogies: An International Journal. (2023) 19:1–18. 10.1080/1554480x.2023.2267038

[35] Rütten A Frahsa A Abel T Bergmann M De Leeuw E Hunter D. Co-producing active lifestyles as whole-system-approach: theory, intervention and knowledge-to-action implications. Health Promot Int. (2019) 34(1):47–59. 10.1093/heapro/dax053 28973298

[36] Biesta G Priestley M Mark R Robinson S. The role of beliefs in teacher agency. Teach Teach. (2015) 21(6):624. 10.1080/13540602.2015.1044325

[37] Biesta G Tedder M. Agency and learning in the lifecourse: towards an ecological perspective. Stud Educ Adults. (2007) 39(2):132. 10.1080/02660830.2007.11661545

[38] Sneck S Syväoja H Järvelä S Tammelin T. More active lessons: teachers’ perceptions of student engagement during physically active maths lessons in Finland. Educ Inq. (2022) 14:458. 10.1080/20004508.2022.2058166

[39] Mogren A Gericke N Scherp HÅ. Whole school approaches to education for sustainable development: a model that links to school improvement. Environ Educ Res. (2019) 25(4):4. 10.1080/13504622.2018.1455074

[40] Morris JL Chalkley A Helme ZE Timms O Young E Mcloughlin GM. Initial insights into the impact and implementation of creating active schools in Bradford, UK. Int J Behav Nutr Phys Act. (2023) 20(1):1. 10.1186/s12966-023-01485-3 37408045 PMC10320983

[41] Craig DW Pfledderer CD Heredia NI Lanza K Onadeko K Pavlovic A. Whole-of-school physical activity promotion: findings from elementary schools in the United States. Am J Prev Med. (2024) 67(6):960–7. 10.1016/j.amepre.2024.08.003 39128591 PMC11585414

[42] Dyngeland ES Resaland GK Mandelid M. Principals’ perceptions on integrating physically active learning through a continuous professional development programme. Teach Teach Educ. (2025) 166:105193. 10.1016/j.tate.2025.105193

[43] Chalkley A Mandelid MB Thurston M Tjomsland HE Morris JL Kallio J. Finding the sweet spot of physically active learning: a need for co-ownership by public health and education. Teach Teach Educ. (2024) 148:104695. 10.1016/j.tate.2024.104695

[44] Mousavi S Gigerenzer G. Heuristics are tools for uncertainty. Homo Oecon. (2017) 34(4):361–79. 10.1007/s41412-017-0058-z

